# A microRNA signature for risk-stratification and response prediction to FOLFOX-based adjuvant therapy in stage II and III colorectal cancer

**DOI:** 10.1186/s12943-022-01699-2

**Published:** 2023-01-20

**Authors:** Keisuke Okuno, Raju Kandimalla, Marta Mendiola, Francesc Balaguer, Luis Bujanda, Carlos Fernandez-Martos, Jorge Aparicio, Jaime Feliu, Masanori Tokunaga, Yusuke Kinugasa, Joan Maurel, Ajay Goel

**Affiliations:** 1 Department of Molecular Diagnostics and Experimental Therapeutics, Beckman Research Institute of City of Hope, Biomedical Research Center, 1218 S. Fifth Avenue, Suite 2226, Monrovia, CA 91016 USA; 2 Department of Gastrointestinal Surgery, Tokyo Medical and Dental University, Tokyo, Japan; 3 Center for Gastrointestinal Research; Center for Translational Genomics and Oncology, Baylor Scott & White Research Institute, Charles A Sammons Cancer Center, Baylor University Medical Center, Dallas, TX USA; 4 Department of Medical Oncology, La Paz University Hospital (IdiPAZ), CIBERONC, cátedra UAM-AMGEN, Madrid, Spain; 5 Department of Gastroenterology, Hospital Clinic de Barcelona, CIBERehd, IDIBAPS, University of Barcelona, Barcelona, Spain; 6 Department of Gastroenterology, Instituto Biodonostia, CIBERehd, Universidad del País Vasco (UPV/EHU), San Sebastián, Spain; 7 Department of Medical Oncology, Hospital Quiron Salud, Valencia, Spain; 8 Department of Medical Oncology, Hospital Universitario y Politécnico La Fe, Valencia, Spain; 9 Translational Genomics and Targeted Therapies Group. Medical Oncology, Hospital Clinic of Barcelona, CIBERehd, IDIBAPS, Villarroel 170, 08036 Barcelona, Spain; 10 City of Hope Comprehensive Cancer Center, Duarte, CA USA

**Keywords:** Colorectal cancer, MicroRNA, Predictive biomarker, Adjuvant chemotherapy, FOLFOX

Clinical decision-making for adjuvant chemotherapy, which includes the selection of the most appropriate patient subgroups and optimal treatment regimens, remains the most pressing challenge in managing patients with stage II and III colorectal cancer (CRC) [1, 2]. For stages III CRC patients, 6 months of FOLFOX-based adjuvant chemotherapy has long been the standard of care treatment following radical surgery of the primary tumor [3–7]; however, given the inherent toxicity of these drugs and inadequate response to these treatments, there is a significant unmet clinical need to identify improved treatment regimens and develop better predictive biomarkers that allow an improved patient selection. Although changes to mono-therapy or shortening the treatment duration from 6 months to 3 months have been attempted in various clinical trials [8, 9], these results are still controversial. Furthermore, 35–40% of stage III patients experience tumor recurrence following adjuvant chemotherapy [3–7]; hence, they are unnecessarily exposed to the toxicity and expense of these drugs without any tangible therapeutic benefit. These patients that do not respond to FOLFOX-based adjuvant therapy could be potential candidates for treatment with more intense adjuvant chemotherapy or other regimens that include some combination of targeted monoclonal therapy or immunotherapy. These data highlight the imperative need to develop predictive biomarkers that can help identify patients in pre-treatment settings who will likely benefit the most from such adjuvant treatments, spare the rest from their toxicity, and are offered alternative individualized treatment strategies.

Herein, we performed a systematic genome-wide microRNA (miRNA) expression profiling to identify and establish a novel miRNA expression signature for predicting response to FOLFOX-based adjuvant chemotherapy in CRC patients. Following biomarker discovery and prioritization, we comprehensively validated a miRNA signature in multiple tissue-based and matched serum-based clinical cohorts and finally established a robust biomarker signature for predicting response to FOLFOX-based chemotherapy in patients with CRC.

## Results and discussions

### Discovery of a 10-miRNA panel from genome-wide small RNA sequencing in patients with and without response to FOLFOX-based chemotherapy

All statistical analyses were performed using EZR version 1.55 [10], a graphical user interface for R version 4.0.3. Two-sided Student’s t-test and Fisher exact test were used for analyzing the difference in continuous and categorical variables, respectively. The cut-off points for continuous variables of clinicopathological factors were divided by the mean value in each clinical cohort.

**Fig. 1:**
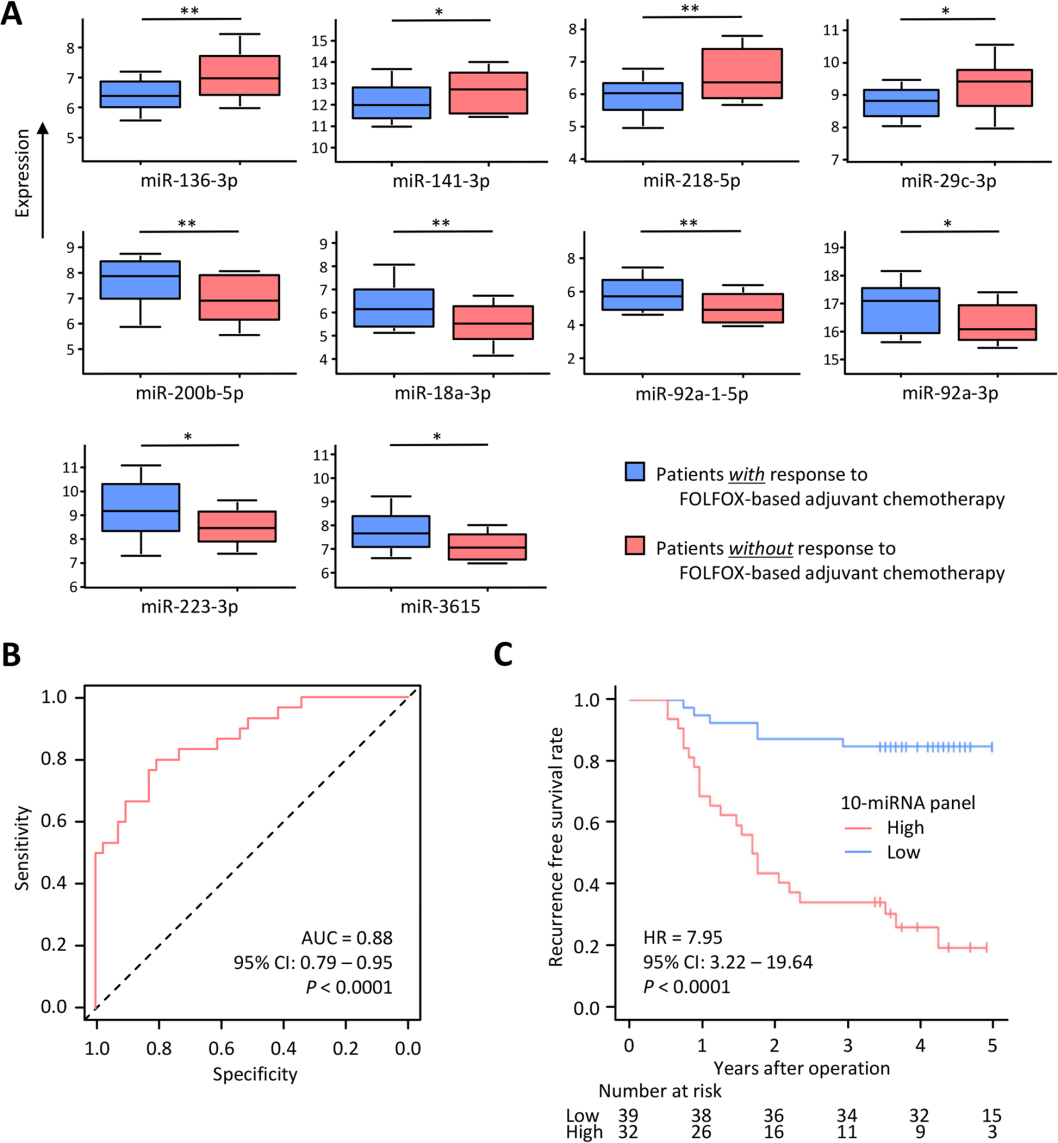
Biomarker discovery phase of candidate miRNAs for predicting response to FOLFOX-based adjuvant chemotherapy in primary tumors from CRC patients. **A** Box plot of 10 candidate miRNAs for predicting the response to FOLFOX in CRC patients. FC and the *P* value of all candidate miRNAs were > 1.5 and < 0.05, respectively. * *P* < 0.05. ** *P* < 0.01. **B** ROC curve values for 10-miRNA panel for predicting response to FOLFOX in the discovery cohort (AUC = 0.88). **C** In discovery cohort, Kaplan-Meier curves of the recurrence-free survival for patients with 10-miRNA panel high (*n* = 32) or low (*n* = 39). ROC, Receiver operating characteristics; AUC, area under the curve; HR, hazard ratio; CI, confidence interval

In the biomarker discovery phase, we analyzed small RNA sequencing profiling data from 71 stages II & III CRC patients (discovery cohort) who were treated with adjuvant FOLFOX therapy (without any targeted monoclonal therapy and immunotherapy) and were enrolled at the University Hospital La Paz, Madrid, Spain. The clinicopathological characteristics of the cohorts are shown in Supplementary Table S1. The raw Fastq files and the processed, filtered count matrix were deposited at the NCBI GEO database under the accession number GSE217978. First, we identified a panel of top 23 miRNAs candidates that were significantly and differentially expressed in patients who developed recurrence (absolute log_2_ fold change [FC] > 1.5 and Benjamini-Hochberg’s corrected, adjusted *P* < 0.05; Supplementary Fig. S1). After that, we narrowed down the list of candidate miRNAs using a LASSO-based Cox regression analysis. We prioritized a panel of 10 miRNAs (4 miRNAs associated with high-risk and six miRNAs associated with low-risk) that were significantly associated with recurrence-free survival (RFS) (Fig. 1A).

We performed the Kaplan-Meier analyses and log-rank tests using the publicly available TCGA dataset to confirm our 10 miRNA biomarkers’ prognostic potential. TCGA dataset was downloaded from the University of California Santa Cruz Xena Browser (https://xenabrowser.net/) and included 172 Stages II & III CRC patients. In these analyses, the overall survival was worse in patients with high expression levels of each of the four miRNAs (miR-136-3p, miR-141-3p, miR-218-5p, and miR-29c-3p) and those with low expression levels of each of the six miRNAs (miR-200b-3p, miR-18a-3p, miR-92a-1-5p, miR-92a-3p, miR-223-3p, and miR-3615) (Supplementary Table S2). Furthermore, when we performed miRNA-target gene and pathway enrichment analyses of these biomarkers using the miRDB [11] and DAVID bioinformatic database [12] to clarify the functional relevance of our biomarkers, many cancer-related pathways were enriched in these miRNAs’ target genes – e.g.*,* ECM-receptor interaction, ERBB signaling, AMPK signaling, PI3K-Akt signaling, TNF signaling, and RAS signaling pathway for four miRNAs associated with high-risk (target score > 80; fold enrichment > 2.0; *P* < 0.01; Supplementary Fig. S2A); Autophagy, ECM-receptor interaction, and Proteoglycan in cancer pathway for six miRNAs associated with low-risk (Supplementary Fig. S2B).

Next, a 10-miRNA panel was generated using the multivariate Cox regression analyses by including all individual miRNAs, and a cut-off threshold of the panel was derived based on Youden’s index [13]. In this initial exploratory cohort, our 10-miRNA panel was significantly correlated with several key clinicopathological factors, including lymphatic and perineural invasion (*P* = 0.01 and 0.04, respectively; Supplementary Table S3). Our 10-miRNA panel demonstrated a promising prognostic potential, as evident from its ability to robustly stratify patients into low and high-risk groups, with 5-year RFS rates of 84.6 and 19.6%, respectively (hazard ratio [HR]: 7.95; 95% confidence interval [CI]: 3.22–19.64; *P* < 0.0001) and a corresponding area under the curve (AUC) value of 0.88 (95% CI: 0.79–0.95; *P* < 0.0001; Fig. 1B and C) in receiver operating characteristics (ROC) curve analyses. Using genome-wide expression profiling in patients with and without response to FOLFOX-based chemotherapy, we identified a novel 10-miRNA expression panel that can robustly predict response to FOLFOX-based adjuvant chemotherapy in patients with CRC.

### Successful validation of the 10-miRNA biomarker panel in an independent tissue- and a serum-based cohort of CRC patients

**Fig. 2:**
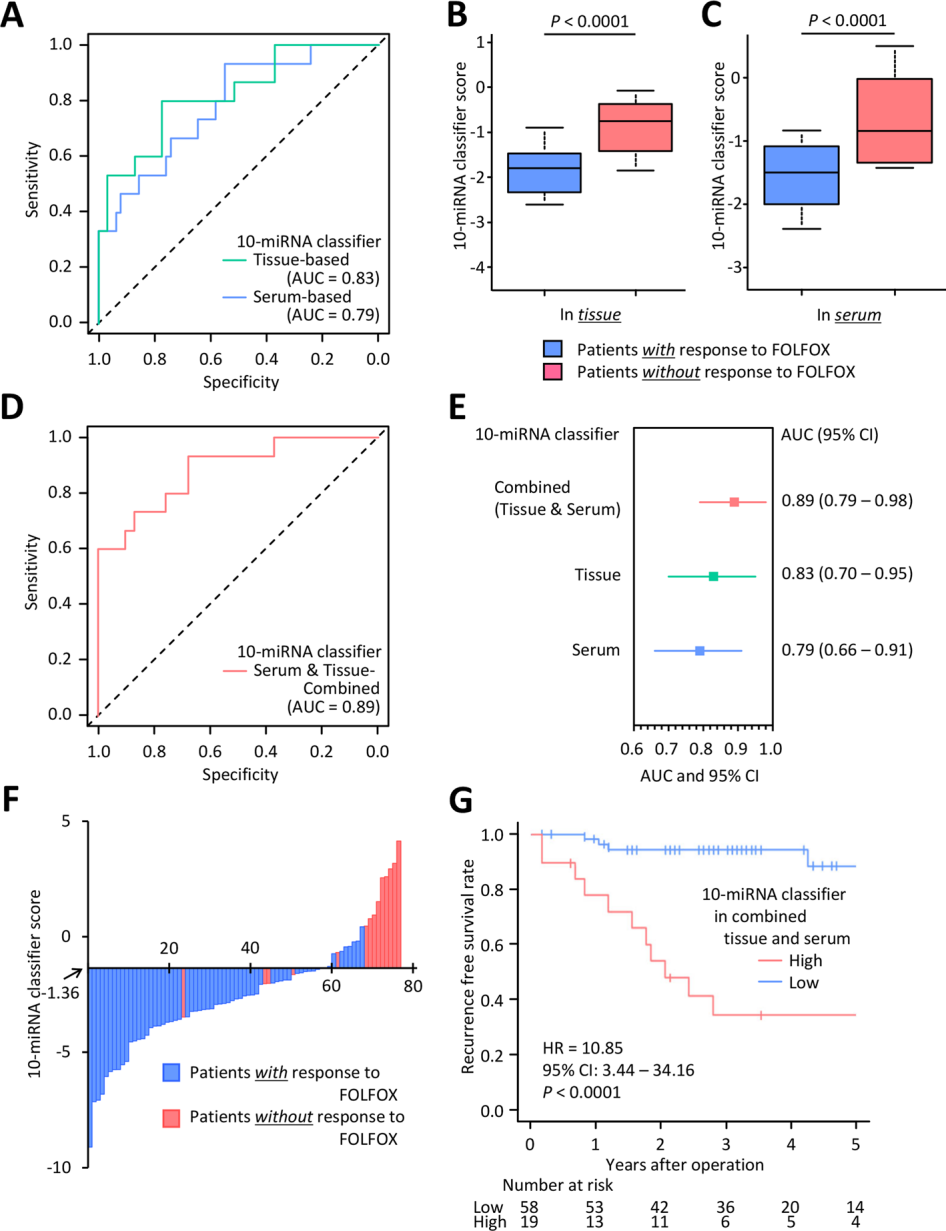
Biomarker validation phase of 10-miRNA classifier for predicting response to FOLFOX-based adjuvant chemotherapy in primary tumors or matched pre-surgery serums from CRC patients of the independent cohort. **A** ROC curve values for 10-miRNA classifier for predicting response to FOLFOX in tissue- and serum-based validation cohort (AUC = 0.83 and 0.79, respectively). **B** Box plot of tissue-based 10-miRNA classifier score for predicting response to FOLFOX in CRC patients. **C** Box plot of serum-based 10-miRNA classifier score for predicting response to FOLFOX in CRC patients. **D** ROC curve values for the combination of tissue- and serum-based 10-miRNA classifier for predicting response to FOLFOX (AUC = 0.89). **E** Forest plot with AUC of combined or tissue-based or serum-based 10-miRNA classifier for predicting response to FOLFOX in CRC patients. **F** Waterfall plot with combined 10-miRNA classifier score for predicting response to FOLFOX in CRC patients. **G** Kaplan-Meier curves of the recurrence-free survival for patients with combined 10-miRNA classifier high (*n* = 19) or low (*n* = 58). CRC, colorectal cancer; ROC, Receiver operating characteristics; AUC, area under the curve; HR, hazard ratio; CI, confidence interval

To validate the prognostic potential of our 10-miRNA panel, we examined the expression of these miRNA markers using quantitative real-time reverse transcription polymerase chain reaction (qRT-PCR) assays in an independent tissue-based cohort of 77 CRC patients enrolled at the University of Donostia San Sebastian, Spain (tissue-based validation cohort). The clinicopathological characteristics of the validation cohorts are shown in Supplementary Table S1, and the raw data of the qRT-PCR assay in the tissue-based cohort are shown in Supplementary Table S4. These experiments aimed to confirm the clinical significance of results and demonstrate the usefulness of simple, inexpensive, and robust qRT-PCR-based assays for an easier translation of these markers in clinical settings. In this validation cohort, a 10-miRNA classifier was generated using the multivariate Cox regression analyses, analogous to our approach in the discovery cohort. A cut-off threshold was derived from Youden’s index [13]. Kaplan-Meier analyses and log-rank tests revealed that the 5-year RFS rates were 89.1% for the miRNA expression-derived high-risk group and 43.4% in the low-risk group (HR: 5.70; 95% CI: 2.87–11.34; *P* < 0.0001), with an AUC of 0.83 (95% CI: 0.70–0.95; *P* = 0.0003) in ROC curve analyses for recurrence prediction (Fig. 2A and Supplementary Fig. S3A). Furthermore, this tissue-based miRNA classifier significantly discriminated against patients with and without response to adjuvant treatment (Fig. 2B).

Additionally, for the performance evaluation as a potential liquid biopsy, we analyzed matched pre-surgery serum specimens from a subset of patients within the tissue-based validation cohort (serum-based validation cohort). The raw data of qRT-PCR assay in a serum-based cohort were shown in Supplementary Table S5. Our 10-miRNA classifier yielded an AUC value of 0.79 (95% CI: 0.66–0.91; *P* = 0.0008; Fig. 2A and C), and Kaplan-Meier analyses and log-rank tests demonstrated that the 5-year RFS rates were 89.1% for the miRNA high-risk group and 50.9% in the low-risk group (HR: 3.20; 95% CI: 1.68–6.07; *P* = 0.0004; Supplementary Fig. S3B). In serum specimens, our 10-miRNA classifier also showed remarkable predictive potential for adjuvant chemotherapy, and these results highlighted the potential of translation to the liquid biopsy assays.

### A combined tissue and serum-based miRNA classifier exhibited superior predictive accuracy for response prediction to FOLFOX-based adjuvant chemotherapy in CRC patients

Although our tissue- and serum-based classifiers exhibited robust predictive accuracies to adjuvant chemotherapy in CRC individually, for their easier clinical translation, we further examined to improve the predictive potential of our classifiers. As multi-omics approaches have recently gained increased attention in clinical oncology practice [14, 15], and tissue and blood specimens are routinely available from stages II and III CRC patients that undergo resection, we hypothesized that a multi-specimen assay might be able to further improve upon the predictive accuracy of our assay. Interestingly, in support of our hypothesis, our combined tissue and serum classifier demonstrated a significantly better performance predicting response to adjuvant chemotherapy, with an AUC of 0.89 (95% CI: 0.79–0.98; *P* = 0.0001; Fig. 2D, E, and F). When we divided all patients into high- or low-risk groups using this combined classifier with a cut-off threshold derived from Youden’s index [13], our combined tissue and serum classifier was significantly correlated with no key clinicopathological factors (Supplementary Table S6), and the 5-year RFS rates were 88.3% for miRNA high-risk group and 34.2% in the low-risk group (HR: 10.85; 95% CI: 3.44–34.16; *P* < 0.0001; Fig. 2G), which was remarkably better discrimination vs. tissue and blood-based marker performance individually.

Furthermore, multivariate Cox regression analyses, including the factors acquired from univariate analysis (*P* < 0.05), revealed that the combined miRNA classifier and carcinoembryonic antigen (CEA) were the significant predictors of RFS in comparison to the clinicopathological variables with an HR of 10.80 (95% CI: 3.40–34.32; *P* < 0.0001) and 4.84 (95% CI: 1.72–13.64; *P* = 0.0028), respectively (Supplementary Table S7). Finally, we compared the performance of our combination, tissue-based, and serum-based classifier for several performance indicators, including sensitivity, specificity, positive predictive value (PPV), negative predictive value (NPV), and predictive accuracy. As expected, our combination classifier demonstrated notably better performance in terms of sensitivity, specificity, PPV, and accuracy (Supplementary Fig. S4). Overall, we successfully developed and established a robust miRNA-based assay to predict the response to adjuvant chemotherapy in CRC patients, and validated this signature in multiple cohorts of stage II and III CRC patients with and without response to FOLFOX-based chemotherapy.

## Conclusions

In the present study, by performing a genome-wide small RNA sequencing and following qRT-PCR validations using tissue and serum specimens from the independent cohort, we succeeded in identifying and establishing a tissue and serum combined 10-miRNA assay that allows the robust prediction of the response to FOLFOX-based adjuvant chemotherapy in stage II and III CRC patients. Our study provides fundamentals of personalized medicine, tailored decision-making in stages II and III CRC patients, and the design of further large sample-size validation in future prospective clinical trials.

## Supplementary Information

**Additional file 1.**

## Data Availability

The data are deposited at the NCBI GEO database under the accession number GSE217978.

## Abbreviations

AUC: Area under the curve
CEA: Carcinoembryonic antigen
CI: Confidence interval
CRC: Colorectal cancer
FC: Fold change
HR: Hazard ratio
miRNAs: microRNAs
MSI: Microsatellite instability
MSS: Microsatellite stability
NPV: Negative predictive value
PPV: Positive predictive value
qRT-PCR: Quantitative real-time reverse transcription polymerase chain reaction
RFS: Recurrence-free survival
ROC: Receiver operating characteristics
