# Supplementary material for: A microRNA signature for risk-stratification and response prediction to FOLFOX-based adjuvant therapy in stage II and III colorectal cancer
Source: Mol Cancer. 2023 Jan 20;22:13. doi: 10.1186/s12943-022-01699-2 (PMC9854096; doi:10.1186/s12943-022-01699-2)
Supplement: Supplementary file 1 — Additional file 1. [file 12943_2022_1699_MOESM1_ESM.pdf]

SUPPLEMENTARY MATERIALS

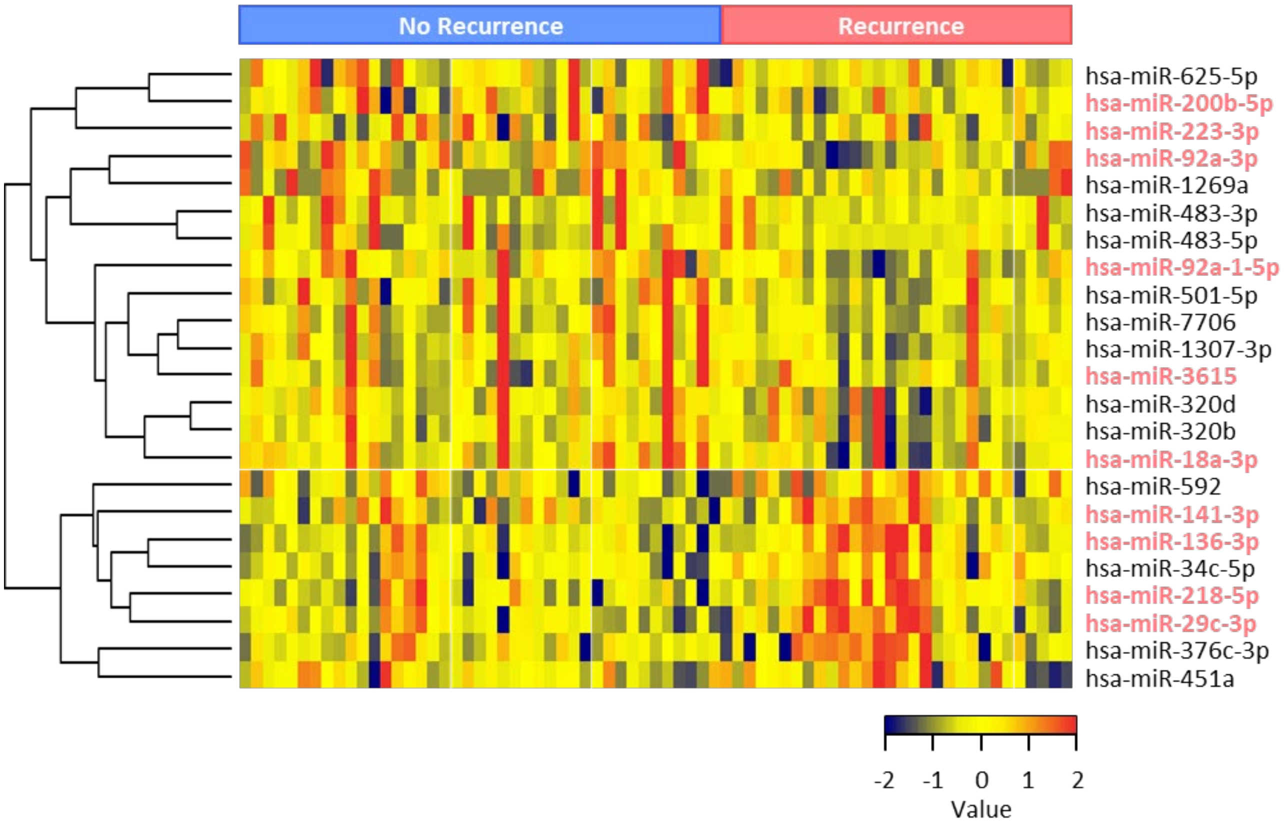

Supplementary Figure S1: Heat map of top 23 miRNAs that are identified from small RNA sequencing. Prioritized miRNAs are highlighted in red.

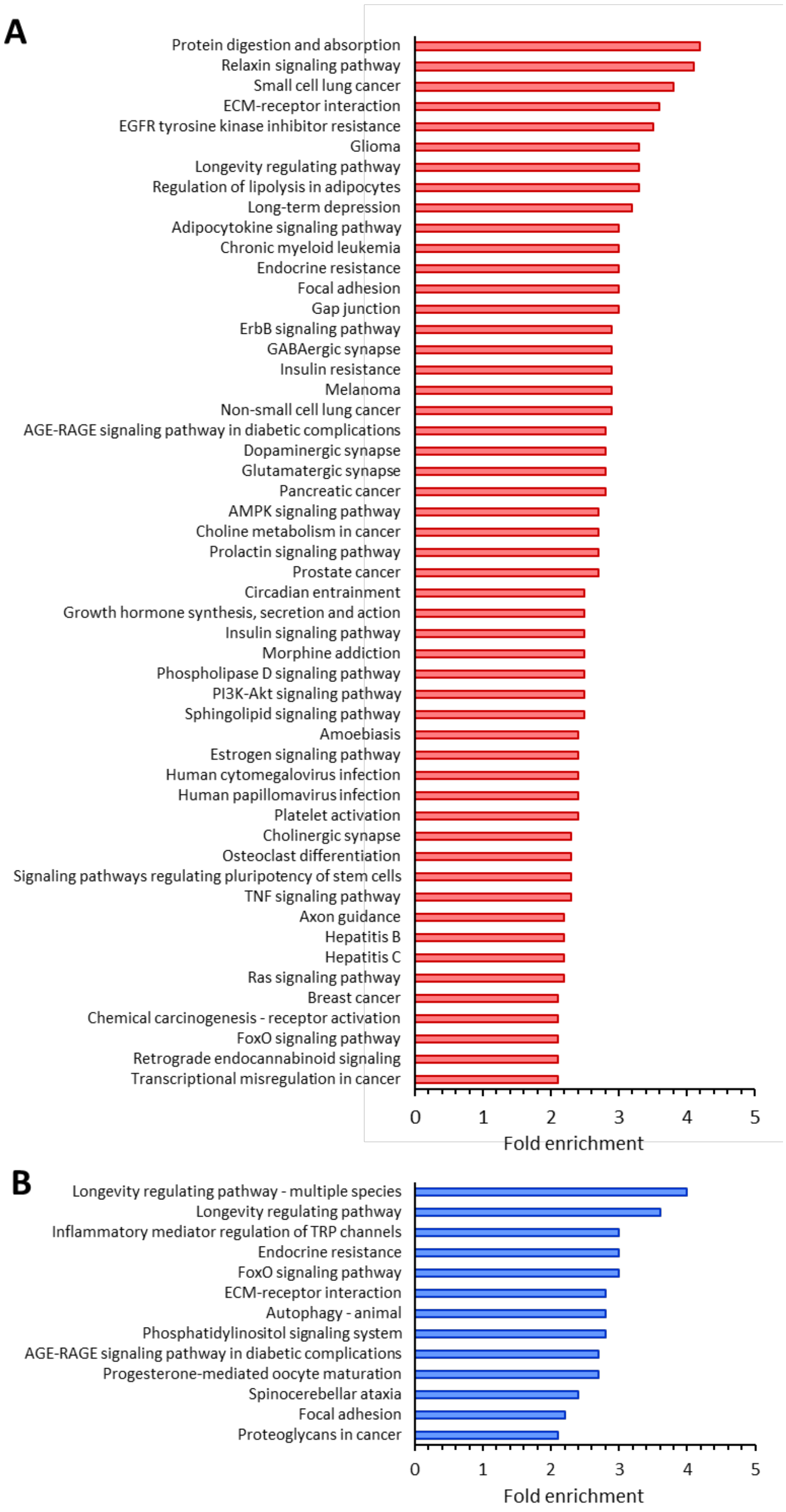

Supplementary Figure S2: The enrichment pathways of 4 upregulated (A) and 6 downregulated (B) miRNA-target genes. A gene was defined as a miRNA-target gene if it had a target score > 80 for each miRNA, and a pathway was defined as enriched if it had $P < 0.01$ and a fold enrichment > 2.0.

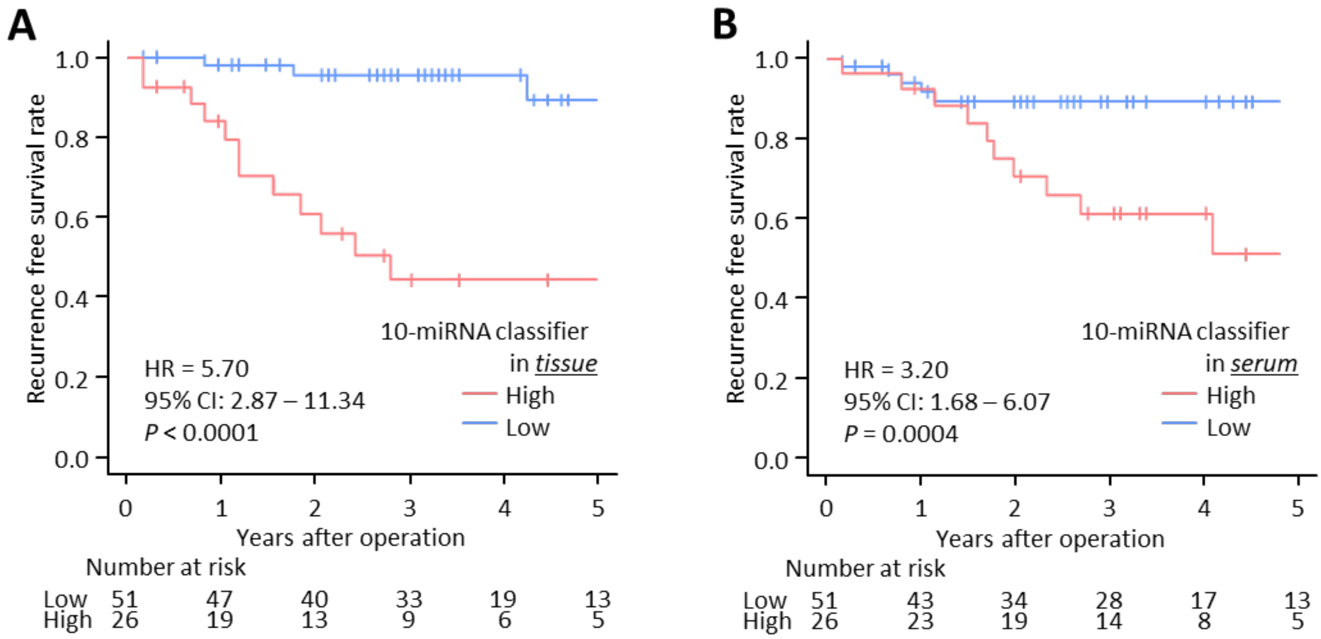

Supplementary Figure S3: Kaplan-Meier curves of the recurrence-free survival for patients with 10-miRNA classifier high or low in tissue- (A) and serum-based (B) validation cohort. HR, hazard ratio; CI, confidence interval.

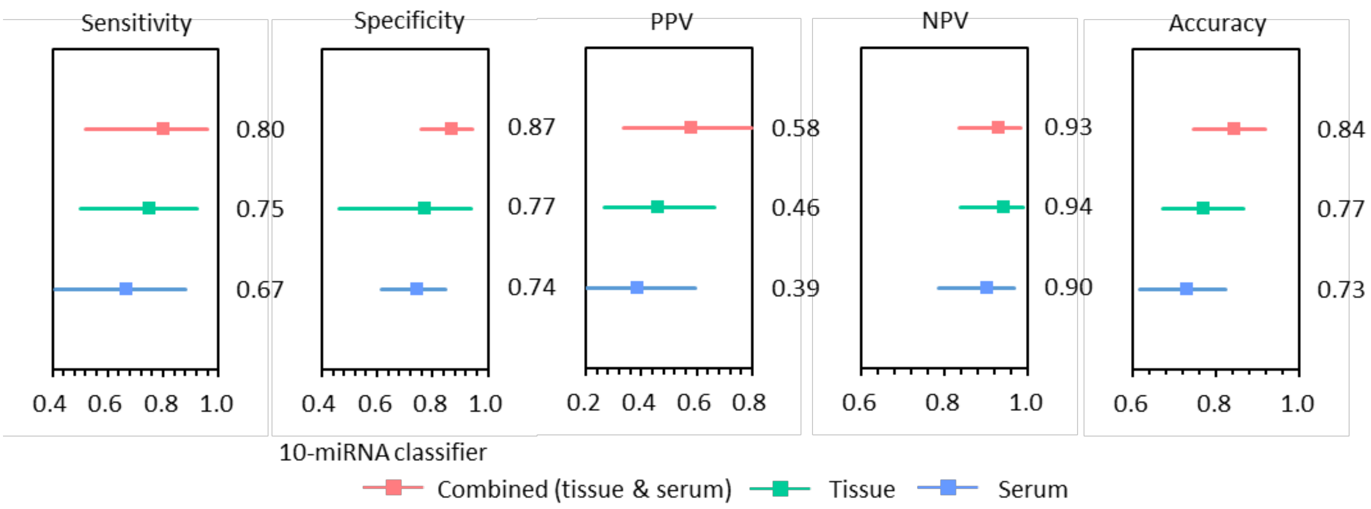

Supplementary Figure S4: Forest plot with sensitivity, specificity, PPV, NPV, and predictive accuracy of combined or tissue-based or serum-based 10-miRNA classifier for the prediction of response to FOLFOX in CRC patients. CRC, colorectal cancer; PPV, positive predictive value; NPV, negative predictive value.

SUPPLEMENTARY TABLE

Supplementary Table S1: Patient's characteristics of each cohort

	Discovery cohort (n = 71)		Validation cohort (n = 77)		*P* value
	n	(%)	n	(%)	
Age, mean (±SD) (years)	63	(±12)	63	(±11)	0.82
Sex					0.18
Male	39	(55)	51	(66)	
Female	32	(45)	26	(34)	
Tumor location					0.57
Right	20	(28)	18	(23)	
Left	51	(72)	59	(77)	
Differentiation					0.45
Well/Moderate	69	(97)	72	(94)	
Poor	2	(3)	5	(6)	
Mucinous differentiation					**0.03**
Negative	71	(100)	71	(92)	
Positive	0	(0)	6	(8)	
Venous invasion					**0.01**
Negative	44	(62)	63	(82)	
Positive	27	(38)	14	(18)	
Lymphatic invasion					0.66
Negative	58	(82)	66	(86)	
Positive	13	(18)	11	(14)	
Perineural invasion					0.26
Negative	49	(69)	60	(78)	
Positive	22	(31)	17	(22)	
T stage					**0.03**
1-2	0	(0)	6	(8)	
3-4	71	(100)	71	(92)	
Lymph node metastases					0.47
Negative	18	(25)	24	(31)	
Positive	53	(75)	53	(69)	
AJCC stage					0.85
II	18	(25)	24	(31)	
III	53	(75)	53	(69)	

MSI status					0.29
High	10	(14)	6	(8)	
Low/MSS	61	(86)	71	(92)	
CEA					0.22
≤ 5.0 ng/ml	60	(85)	58	(75)	
> 5.0 ng/ml	11	(15)	19	(25)	

SD, standard deviation; AJCC, the American Joint Committee on Cancer; MSI, microsatellite instability; MSS, microsatellite stability; CEA, carcinoembryonic antigen

Supplementary Table S2: Kaplan-Meier analyses and log-rank tests of each miRNA biomarker in TCGA dataset

	**Cut-off value** by Youden index	**HR** (high level vs. low level)	**95% CI**	***P* value**
miR-136-3p	2.88	2.17	1.12-4.23	**0.02**
miR-141-3p	9.45	1.56	0.79-3.09	0.20
miR-218-5p	4.25	1.93	1.01-3.70	**0.04**
miR-29c-3p	9.73	2.44	1.20-4.96	**0.01**
miR-200b-5p	6.28	0.55	0.28-1.06	0.07
miR-18a-3p	1.61	0.58	0.30-1.11	0.10
miR-92a-1-5p	2.73	0.86	0.44-1.66	0.65
miR-92a-3p	14.11	0.41	0.21-0.79	**< 0.01**
miR-223-3p	7.75	0.77	0.41-1.48	0.44
miR-3615	1.92	0.63	0.33-1.20	0.16

HR, hazard ratio; CI, confidence interval

Supplementary Table S3: Clinicopathological correlation of 10-miRNA panel in the discovery cohort

	**10-miRNA panel low** (n = 39)		**10-miRNA panel high** (n = 32)		*P* value
	n	(%)	n	(%)	
Age, mean (±SD) (years)	61	(±13)	65	(±10)	0.12
Sex					0.63
Male	20	(51)	19	(59)	
Female	19	(49)	13	(41)	
Tumor location					0.31
Right	13	(33)	7	(22)	
Left	26	(67)	25	(78)	
Differentiation					1.00
Well/Moderate	38	(97)	31	(97)	
Poor	1	(3)	1	(3)	
Venous invasion					1.00
Negative	24	(62)	20	(63)	
Positive	15	(39)	12	(38)	
Lymphatic invasion					**0.01**
Negative	36	(92)	22	(69)	
Positive	3	(8)	10	(31)	
Perineural invasion					**0.04**
Negative	31	(80)	18	(56)	
Positive	8	(21)	14	(44)	
T stage					NA
1-2	0	(0)	0	(0)	
3-4	39	(100)	32	(100)	
Lymph node metastases					0.59
Negative	11	(28)	7	(22)	
Positive	28	(72)	25	(78)	
AJCC stage					0.59
II	11	(28)	7	(22)	
III	28	(72)	25	(78)	
MSI status					0.50

High	7	(18)	3	(9)	
Low/MSS	32	(82)	29	(91)	
CEA					0.53
≤ 5.0 ng/ml	34	(87)	26	(81)	
> 5.0 ng/ml	5	(13)	6	(19)	

SD, standard deviation; AJCC, the American Joint Committee on Cancer; MSI, microsatellite instability; MSS, microsatellite stability; CEA, carcinoembryonic antigen

Supplementary Table S4: The raw data of qRT-PCR assay in tissue-based validation cohort

Sample Number	miR-136-3p	miR-141-3p	miR-18a-3p	miR-200b-5p	miR-218-5p	miR-223-3p	miR-29c-3p	miR-3615	miR-92a-1-5p	miR-92a-3p
1	-0.67393	2.377551	0.627702	0.707767	0.02078	0.785688	2.256975	-0.95721	0.362404	2.417759
2	-0.14383	2.947241	0.948821	1.018337	0.240757	1.613753	2.719661	-0.36194	1.012221	2.672477
3	-0.2084	2.871845	1.097277	1.199072	0.656662	2.1539	2.692129	-0.52868	1.408519	2.722953
4	-0.07282	2.48101	0.673519	0.668195	0.927303	1.308159	2.537264	-0.70772	-0.59332	2.371374
5	-0.59817	1.673391	1.230427	1.331363	1.058494	1.687658	2.663195	-0.7884	-0.25908	3.052989
6	-0.65412	2.888597	1.066195	0.99969	-0.00208	1.080947	2.739987	-0.89847	-0.77812	2.762195
7	-0.81726	1.972651	0.78412	0.574086	-0.09869	1.28827	2.504866	-1.08768	-0.54406	2.514443
8	0.242565	3.43677	2.005424	1.63674	1.151509	2.363191	3.537911	0.234441	0.418987	3.248065
9	-0.32389	2.438274	1.150105	0.733429	0.882871	1.681609	2.68854	-0.87476	-0.45381	2.60334
10	-0.68499	2.697567	1.279741	0.50961	0.532488	1.1322	2.458411	-0.7329	-0.86956	2.532633
11	-0.5781	2.722244	1.368096	1.027717	0.658637	1.817855	2.68854	-0.57756	-0.57764	2.572157
12	-0.47835	3.124874	1.263855	1.267395	0.179349	1.452745	3.040303	-0.5323	-0.01526	3.2036
13	-0.31144	2.958813	0.978002	1.024778	0.635173	1.905216	2.720903	-0.54988	-0.57111	2.353597
14	-0.49347	3.08117	1.395359	1.367937	0.909851	1.311896	2.822126	-0.678	-0.12859	2.896194
15	-2.16618	-0.02979	-0.99666	-1.91358	-1.7232	0.294451	0.659528	-2.42738	-2.70796	0.49875
16	-0.61545	2.334786	0.551022	0.786664	0.13941	1.222682	2.442487	-1.17897	-0.92082	2.352465
17	-0.28965	2.73743	1.215658	0.810715	0.924018	2.337715	3.039631	-0.26522	-0.56782	2.843012
18	-0.70742	2.63365	0.903867	1.086152	0.735485	1.466912	2.646226	-0.83006	-0.56663	2.525415
19	-0.75819	2.37568	0.613079	0.948641	-0.17696	1.219361	2.300558	-0.96882	-0.92437	2.193064
20	-0.56975	2.399696	0.576092	0.762984	0.572426	1.526496	2.643115	-0.73071	-0.85188	2.149084
21	-0.454	2.581425	1.294645	1.081246	0.903282	1.755808	2.538439	-0.42906	-0.28009	2.626481
22	-0.6824	2.093064	0.926218	0.501193	-0.074	1.300112	2.442258	-1.20196	-0.69322	2.314537
23	-0.15104	3.735541	1.672217	1.961345	0.976158	2.007946	3.357159	0.074285	0.094715	3.387068
24	0.439643	3.504309	1.724338	1.503062	0.924931	2.29287	3.651876	0.542789	-0.17129	3.531554
25	0.4927	3.572209	2.127074	2.170071	1.781541	2.568624	3.722934	0.102997	0.544874	3.808738
26	-0.5733	2.259761	1.101835	0.730302	-0.18406	1.49937	2.355235	-0.49211	-0.44897	2.524234
27	-1.17706	2.738168	1.256234	1.318578	-0.24064	0.594227	2.262968	-0.88711	0.050171	3.024439
28	-0.57273	2.530039	0.952196	0.703015	0.530463	1.230073	2.474824	-0.88368	-0.80479	2.43072
29	-0.51018	2.441388	0.753819	0.593878	0.344574	0.928167	2.574975	-0.79979	-0.90161	2.412071
30	-0.45665	2.494017	0.379004	0.440241	0.429613	1.057034	2.304953	-1.1117	-0.96012	2.015745
31	-0.26408	2.875749	1.620552	1.186931	0.60083	2.016668	2.776474	-0.40282	-0.06588	2.750995
32	-1.00479	2.827009	1.025676	1.139296	0.264247	1.253384	2.734217	-0.69917	-0.48558	2.757803
33	-1.94003	1.560447	0.446942	-0.08055	-1.57852	0.293698	1.540436	-1.82218	-1.3277	1.749231
34	-0.72261	2.587131	1.299104	0.886014	-0.09774	1.080292	2.765653	-0.82875	-0.36738	2.747871

35	-1.10585	1.951896	0.471074	0.41222	-0.32123	0.895757	1.78484	-1.27735	-1.16746	1.915983
36	-0.47811	1.624973	0.102359	0.067335	0.55456	1.795432	2.067703	-0.89736	-1.49817	1.810462
37	1.0833	3.325565	1.177655	1.456477	2.065502	2.614353	2.931046	0.06884	-0.02372	2.742641
38	0.343309	2.981611	1.077155	1.429937	1.818216	2.224441	2.746109	-0.22904	0.033893	2.92778
39	-0.08175	3.116394	1.862586	1.181989	1.09616	1.61452	2.626154	-0.5459	-1.05692	2.531013
40	-1.60627	1.65506	0.038481	-0.25961	-0.86656	0.610753	1.131908	-1.78121	-1.9452	1.6506
41	-1.41629	1.589159	0.242198	-0.16302	-1.12228	0.018317	1.454987	-1.93937	-1.3993	1.673802
42	-0.17368	2.568783	0.930013	0.869434	1.17546	1.396788	2.881412	-0.90104	-0.7316	2.596509
43	-0.05208	2.386555	1.444785	1.681402	1.110793	2.008768	3.135738	-0.3308	0.101447	3.057006
44	-1.29423	2.296008	-0.31304	-0.3632	-0.81206	2.196975	1.769875	-1.57399	-2.00297	0.972443
45	-0.7438	3.178014	1.109331	1.533706	0.413577	2.180573	3.111872	-0.34924	0.044458	2.606429
46	0.006423	2.756063	1.353212	1.065141	1.178533	1.9804	2.932891	-0.27207	-0.14663	2.530631
47	-1.69716	1.681582	0.746198	0.079459	-0.42699	0.383046	1.780541	-1.57471	-0.70462	2.002084
48	-0.96609	2.092535	0.617662	0.340926	-0.18588	0.849553	2.345553	-1.02237	-0.94844	2.019611
49	-0.38027	2.555057	0.669893	0.780509	0.467869	1.524464	2.224295	-0.65732	-0.90159	2.316577
50	0.219308	3.141728	1.123825	1.448988	0.981222	1.709171	3.018484	-0.11245	-0.04219	2.732443
51	-0.57284	2.226616	0.063357	0.330008	0.084372	0.788477	1.900336	-1.28868	-0.93046	1.866896
52	0.664807	3.67465	1.907329	1.828649	1.705745	2.934974	3.787205	0.482125	0.252296	3.36692
53	-1.19239	1.736073	0.42011	-0.00594	-0.38876	1.007671	2.14073	-1.26305	-1.29455	2.120974
54	-1.00558	2.110232	0.69319	0.610773	-0.31305	0.86645	2.128823	-0.84966	-0.97799	2.30359
55	0.422088	3.136952	1.57955	1.535978	1.617541	2.565263	3.214431	-0.09203	-0.52705	3.257397
56	-1.22916	2.466443	0.943661	0.507785	-0.41945	0.859474	2.253683	-0.81615	-0.91882	2.471959
57	-0.4666	2.885849	1.078994	0.939616	0.74169	2.140532	2.808995	-0.70823	-0.64105	2.914182
58	-0.9574	2.306248	0.197079	0.404349	-0.2283	0.768594	2.315241	-1.05062	-0.52078	2.604787
59	-0.66425	2.526959	0.424003	0.60329	0.523316	1.051207	2.816831	-1.31368	-0.38253	2.84625
60	-1.00799	1.690222	-0.34006	-0.20556	0.564025	0.651108	2.286745	-1.55896	-2.07873	1.740083
61	-0.58073	1.955789	0.917718	0.461135	0.203237	1.176561	2.363994	-0.93496	-0.73229	2.589729
62	-0.07053	2.47913	1.096395	0.931979	0.735117	2.312443	2.948555	-0.76201	-0.66888	2.379676
63	-1.12014	2.282291	0.720937	0.475181	-0.4482	1.338129	2.311397	-0.78517	-1.3824	1.783292
64	0.630405	3.826446	1.796183	1.924531	1.521153	2.32821	3.997582	0.064681	0.435205	3.500046
65	-0.68896	2.179711	0.418348	0.336407	-0.15018	0.848296	2.207451	-0.90758	-0.9057	2.117531
66	-0.33642	2.340634	0.195934	0.629811	0.960138	1.614997	2.380039	-0.75284	-0.99968	2.098167
67	-0.71443	2.581509	1.042322	0.96699	-0.04051	1.429565	2.487504	-0.6902	-0.53852	2.33632
68	-0.93616	1.899347	0.141905	0.041539	-0.20758	0.522885	2.255099	-1.28056	-1.30529	1.954196
69	-0.77584	1.842941	0.295088	0.365669	0.019152	1.075919	1.954742	-1.3143	-1.20101	2.000609
70	-0.51467	2.640341	0.584304	0.928672	0.437821	1.227098	2.474619	-0.74472	-1.1165	2.121844
71	-0.68798	2.784852	1.09209	0.703059	0.140636	0.866839	2.801122	-1.03733	-0.21809	2.695986
72	-1.25629	2.305697	0.320911	0.741039	-0.93669	1.549203	2.208874	-0.83161	-1.26677	1.978074

73	-0.73005	1.892447	0.512989	-0.02205	0.67569	1.188721	2.473227	-1.25863	-0.99294	2.450058
74	-1.40554	1.95507	0.612177	-0.10971	-0.49221	1.785675	1.925221	-1.05643	-1.3016	1.879175
75	-0.35459	2.827569	0.895172	0.870694	1.012753	1.93354	2.791302	-0.81145	-0.49963	2.601932
76	-1.4638	2.075112	0.709094	0.268384	-0.53197	0.618448	1.916404	-0.82971	-0.77297	2.438764
77	-0.93492	1.584097	0.073795	-0.0363	0.334619	1.017026	2.023537	-1.61744	-1.72597	1.729495

Supplementary Table S5: The raw data of qRT-PCR assay in serum-based validation cohort

Sample Number	miR-136-3p	miR-141-3p	miR-18a-3p	miR-200b-5p	miR-218-5p	miR-223-3p	miR-29c-3p	miR-3615	miR-92a-1-5p	miR-92a-3p
1	-2.08067	-2.0899	-0.79532	-2.47803	-1.84297	1.557255	-0.29279	-1.23202	-2.4917	1.228725
2	-2.25646	-2.1016	-0.67327	-2.62696	-2.52954	1.821623	-0.32375	-1.03345	-2.5466	1.308416
3	-1.81851	-1.5109	-0.50897	-2.03062	-1.79467	1.645363	-0.04933	-1.07367	-4.0030	2.033947
4	-1.96212	-1.9596	-1.01484	-2.78541	-3.27137	1.770759	-0.21698	-1.09419	-2.3504	0.963198
5	-1.8699	-1.1579	-0.70299	-2.00034	-1.63246	1.328855	-0.08203	-1.02904	-3.5242	1.745597
6	-1.47189	-1.8831	-0.61146	-2.60855	-2.48952	1.57342	-0.19752	-1.07762	-2.1207	1.466434
7	-3.92526	-1.9763	-0.84233	-1.73072	-2.05728	1.3983	-0.31801	-1.17305	-1.9137	1.406202
8	-4.67346	-2.0498	-0.67763	-2.50552	-3.06981	1.487377	-0.34219	-0.98464	-2.3635	1.359757
9	-1.91159	-1.3409	0.063113	-1.22305	-1.88229	2.616257	0.119783	-1.08712	-3.5185	2.168128
10	-2.04766	-1.4231	-0.49161	-2.02405	-2.32944	2.333294	0.019155	-0.9106	-4.5886	1.491908
11	-1.91728	-1.9819	-0.88993	-2.47193	-3.95187	1.171459	-0.56989	-1.56721	-2.3464	1.371062
12	-1.86506	-1.1998	-0.62035	-1.76455	-2.38313	2.088947	0.009769	-1.02701	-2.0804	1.483541
13	-2.28708	-1.8920	-0.97179	-2.45942	-3.77443	1.204878	-0.46088	-1.6746	-2.0341	1.214132
14	-2.93685	-1.3671	0.061521	-0.9622	-2.93685	2.438006	0.034482	-0.96637	-2.9368	2.33127
15	-2.53919	-1.9901	-0.56353	-4.29095	-3.8934	1.930124	-0.3077	-1.35858	-2.0464	1.560493
16	-3.16778	-2.5196	0.084109	-0.96001	-3.16778	2.393915	0.214344	-1.18836	-3.1677	2.745339
17	-1.28835	-3.2466	0.381654	-2.60724	-1.65391	2.608956	0.303932	-0.69712	-1.3557	3.255386
18	-1.32611	-1.2085	-0.18147	-0.94172	-1.31835	2.366805	0.324732	-0.57604	-3.2439	2.437387
19	-2.83262	-0.6556	-0.13379	-1.01624	-1.49299	2.441128	0.562261	-1.00535	-0.9966	2.355657
20	-1.71888	-1.7199	0.526843	-2.24189	-2.93759	1.51126	0.57961	-0.29054	-2.2957	2.968056
21	-2.01202	-2.0006	-0.67021	-1.80398	-3.6186	1.722946	-0.21259	-1.22641	-1.6308	1.637671
22	-2.4498	-2.0212	-0.87464	-2.38879	-2.32434	1.60175	-0.45109	-1.64214	-1.9509	1.399142
23	-2.0318	-1.7179	-0.44129	-1.79187	-2.99979	1.728789	-0.27576	-1.22416	-1.4487	1.886591
24	-3.80705	-1.9780	-0.15798	-1.63805	-1.29739	2.633877	0.243564	-0.95937	-1.7390	2.029155
25	-1.80443	-2.1875	-0.50704	-1.4978	-1.72966	2.101879	-0.07673	-1.20758	-1.6055	1.962672
26	-2.94517	-1.8668	-0.47965	-1.2682	-1.78201	1.691052	-0.11816	-1.26467	-3.6180	2.027616
27	-1.87975	-2.1151	-0.98172	-1.80682	-2.47838	1.464523	-0.53883	-1.62862	-1.3217	1.767764
28	-2.54364	-2.5436	0.395698	-0.43043	-2.54364	2.37118	0.563604	-0.34993	-2.5436	3.1861
29	-2.31025	-2.3102	-0.19885	-0.68023	-2.31025	2.095203	0.050922	-0.7982	-1.8843	2.777083
30	-0.50281	-2.4917	0.482168	-1.03145	-0.54341	2.809508	1.207299	-0.71366	-2.4917	3.198658
31	-1.78738	-1.7873	0.329567	-1.75624	-1.78738	3.085297	0.513824	-0.48485	-1.7873	3.287341
32	-2.61318	-1.2359	0.07306	-1.3499	-2.61318	2.385213	0.315598	-0.71987	-2.6131	2.948272
33	-1.45524	-2.2824	-0.71921	-1.93199	-2.15477	1.482068	-0.34363	-1.30802	-2.7598	1.617424

34	-2.01041	-1.7048	-0.60617	-2.35449	-2.12465	1.352947	-0.32939	-1.17851	-2.3138	1.795405
35	-1.35714	-2.053	-0.89715	-2.36503	-2.43512	1.277541	-0.46618	-1.26653	-2.3491	1.542013
36	-1.54809	-1.3127	-0.28463	-2.11244	-1.32151	2.097204	0.248227	-0.7585	-3.2366	2.675753
37	-0.60295	-1.1247	0.27365	-0.96877	-2.8637	2.48536	0.464541	-0.48986	-0.9767	3.093691
38	-1.33544	-1.3354	0.912606	0.240987	0.135935	3.032837	1.075992	0.335205	-1.3354	3.634176
39	-0.55223	-2.4485	0.796083	-0.63343	-0.47792	2.529743	0.91614	-0.15366	-1.0765	3.512675
40	-1.9669	-1.5713	0.537666	-1.27344	-1.2165	2.24102	0.605728	-0.84452	-1.0573	2.768796
41	-2.18249	-1.9831	-0.61016	-1.95209	-1.34804	0.934871	-0.29263	-1.10654	-2.3605	1.841227
42	-1.32587	-1.9009	-0.95469	-1.70349	-1.71718	0.798165	-0.33185	-1.34705	-2.3387	1.496946
43	-1.86593	-2.0429	-1.04564	-1.93013	-2.95626	0.988362	-0.5178	-1.35364	-2.6415	1.370274
44	-0.70968	-0.9532	-0.32361	-1.07832	-1.87812	1.909661	0.31029	-0.59607	-3.1956	2.645833
45	-1.76392	-4.5990	-1.04393	-2.37166	-3.01177	0.874578	-0.52122	-1.32222	-2.4570	1.458202
46	-3.25808	-1.4406	-0.08406	-1.19243	-3.25808	1.958368	0.200647	-0.81428	-3.2580	2.394539
47	-0.51941	-1.9046	0.006464	-0.53796	-1.9046	2.156999	0.001681	-0.52215	-1.9046	2.787903
48	-1.90317	-0.5871	-0.01597	-0.54883	-1.90317	1.58711	0.292672	-0.38445	-1.9031	2.496133
49	-2.0257	-1.1677	-0.06056	-0.17185	-2.0257	2.308297	0.396231	-0.11403	-2.0257	2.928999
50	-1.83111	-1.8311	0.345656	-0.20581	-1.83111	1.493842	0.334227	-0.15856	-0.2367	3.024495
51	0.097881	-0.6208	0.654087	0.137849	-1.55238	2.450223	0.739478	0.007984	-1.5523	3.570897
52	-2.30507	-2.1980	-0.08784	-0.45071	-0.38166	2.142568	0.751163	-0.36325	-2.3050	2.888044
53	-2.10501	-1.9581	-0.81622	-1.414	-2.36172	1.059128	-0.21567	-1.41372	-2.0081	1.55284
54	-2.49757	-1.8141	-0.74422	-1.5965	-2.00322	1.27369	-0.33514	-1.28535	-1.9140	1.737389
55	-2.67401	-2.3415	-0.27802	-2.5445	-2.9071	0.574053	-0.06479	-1.11551	-2.6946	1.883514
56	-2.27374	-2.2942	-0.49972	-2.50813	-3.20769	0.768528	-0.39027	-1.31061	-2.9866	1.488896
57	-1.24951	-2.3694	-0.49577	-1.12513	-2.36942	2.152917	0.109332	-0.87731	-2.3694	2.97792
58	-0.96649	-1.2576	0.728797	-0.70613	-0.78528	2.520282	0.833278	-0.30933	-2.6907	3.535564
59	-3.74967	-1.7437	0.13558	-1.37059	-1.812	2.280566	0.285569	-0.94153	-1.3347	2.646135
60	-2.30974	-2.3097	0.443967	-0.3278	-0.67191	2.351975	0.443512	-0.71134	-2.3097	3.244939
61	-2.05975	-1.8482	0.244694	-1.38508	-1.74159	2.265624	0.448282	-0.75037	-1.6712	2.923529
62	-2.24223	-1.8868	-0.41997	-2.86357	-2.52749	0.90793	0.241974	-1.42691	-2.6943	1.705844
63	-2.31353	-2.3135	0.226697	-0.67489	-0.26513	1.956328	0.208498	-0.51118	-2.3135	2.988224
64	-2.0032	-2.0692	-0.79895	-2.04465	-4.90807	0.904097	-0.23492	-1.18597	-2.3784	1.710197
65	-1.72477	-4.1399	-1.23026	-1.90877	-2.50826	1.152308	-0.78794	-1.88782	-4.1399	1.480433
66	-1.87818	-2.0234	-1.06097	-2.26273	-2.8877	1.184738	-0.29523	-1.41688	-2.4617	1.286468
67	-2.01739	-2.3108	-1.08194	-2.86482	-3.30771	0.830416	-0.66213	-1.58664	-2.2099	1.270216
68	-1.50231	-2.0651	-0.31871	-1.68469	-1.12676	1.821957	0.136582	-1.0514	-1.6792	2.368576
69	-0.97871	-2.6944	0.151701	-0.56214	-0.53637	2.039626	0.567141	-0.36899	-2.6944	3.230939
70	-1.1486	-1.1486	0.314082	0.58819	-1.1486	2.110351	0.439277	0.091306	-1.1486	3.276403
71	-2.79338	-2.7933	0.307265	-0.76282	-0.82143	2.18154	0.172292	-0.56239	-2.7476	3.344586

72	-2.39086	-2.3908	0.093057	-0.2738	-0.18527	3.193161	0.671449	-0.10629	-2.3908	3.192911
73	-1.88663	-2.0708	-0.96677	-1.89913	-4.07288	1.434372	-0.33844	-1.31248	-2.1857	1.754084
74	-1.77247	-2.1832	-1.28426	-2.24951	-4.72862	0.960141	-0.71343	-1.41816	-2.7755	1.284531
75	-0.30293	-2.0377	-0.34552	-1.0439	-2.03779	2.146551	0.277403	-0.3254	-2.0377	3.278734
76	-3.2221	-1.8667	0.1051	-2.08963	-2.31461	1.612072	0.177583	-0.41778	-2.3716	2.611275
77	-1.73416	-2.0672	-1.30375	-2.25895	-4.70234	0.657584	-0.92371	-1.65992	-2.2692	1.009343

Supplementary Table S6: Clinicopathological correlation of combined tissue and serum-based miRNA classifier in the validation cohort

	Combined miRNA classifier low (n = 58)		Combined miRNA classifier high (n = 19)		*P* value
	n	(%)	n	(%)	
Age, mean (±SD) (years)	62	(±11)	65	(±13)	0.29
Sex					1.00
Male	38	(66)	13	(68)	
Female	20	(35)	6	(32)	
Tumor location					1.00
Right	14	(24)	4	(21)	
Left	44	(76)	15	(79)	
Differentiation					1.00
Well/Moderate	54	(93)	18	(95)	
Poor	4	(7)	1	(5)	
Mucinous differentiation					0.63
Negative	54	(93)	17	(90)	
Positive	4	(7)	2	(11)	
Venous invasion					0.10
Negative	50	(86)	13	(68)	
Positive	8	(14)	6	(32)	
Lymphatic invasion					0.13
Negative	52	(90)	14	(74)	
Positive	6	(10)	5	(26)	
Perineural invasion					0.34
Negative	47	(81)	13	(68)	
Positive	11	(19)	6	(32)	
T stage					1.00
1-2	5	(9)	1	(5)	
3-4	53	(91)	18	(95)	
Lymph node metastases					0.39
Negative	20	(35)	4	(21)	
Positive	38	(66)	15	(79)	

AJCC stage					0.57
II	18	(31)	6	(32)	
III	40	(69)	13	(68)	
MSI status					1.00
High	5	(9)	1	(5)	
Low/MSS	53	(91)	18	(95)	
CEA					0.54
≤ 5.0 ng/ml	45	(78)	13	(68)	
> 5.0 ng/ml	13	(22)	6	(32)	

SD, standard deviation; AJCC, the American Joint Committee on Cancer; MSI, microsatellite instability; MSS, microsatellite stability; CEA, carcinoembryonic antigen

Supplementary Table S7: Univariate and multivariate analysis of factors contributing to recurrence free survival in validation cohort

	**Univariate**		**Multivariate**	
	HR (95% CI)	*P* value	HR (95% CI)	*P* value
Age, > 63 versus ≤ 63 years	1.06 (0.38 - 2.99)	0.91		
Sex, male versus female	1.13 (0.38 - 3.29)	0.83		
Tumor location, left versus right	2.11 (0.48 - 9.36)	0.33		
Differentiation, poor versus well and moderate	NA	NA		
Venous invasion, positive versus negative	1.76 (0.56 - 5.52)	0.34		
Lymphatic invasion, positive versus negative	1.63 (0.46 - 5.77)	0.45		
Perineural invasion, positive versus negative	1.71 (0.58 - 4.99)	0.33		
T stage, T3-4 versus T1-2	1.26 (0.17 - 9.62)	0.82		
Lymph node metastasis, positive versus negative	2.32 (0.65 - 8.22)	0.19		
MSI status, low and MSS versus high	NA	NA		
CEA, > 5.0 versus ≤ 5.0 ng/ml	4.91 (1.77 - 13.58)	**< 0.01**	4.84 (1.72 - 13.64)	**< 0.01**
10-miRNA classifier of combination, high versus low	10.85 (3.44 - 34.16)	**< 0.01**	10.80 (3.40 - 34.32)	**< 0.01**

HR, hazard ratio, CI, confidence interval, MSI, microsatellite instability, MSS, microsatellite stability, CEA, carcinoembryonic antigen
